# Allogeneic Hematopoietic Stem Cell Transplantation Mobilized With Pegylated Granulocyte Colony-Stimulating Factor Ameliorates Severe Acute Graft-Versus-Host Disease Through Enrichment of Monocytic Myeloid-Derived Suppressor Cells in the Graft: A Real World Experience

**DOI:** 10.3389/fimmu.2021.621935

**Published:** 2021-04-12

**Authors:** Lin Li, Jin Yin, Yun Li, Chunyan Wang, Xia Mao, Jia Wei, Yang Cao, Na Wang, Li Lin, Jinhuan Xu, Yicheng Zhang

**Affiliations:** ^1^ Department of Hematology, Tongji Hospital, Tongji Medical College, Huazhong University of Science and Technology, Wuhan, China; ^2^ Institute of Organ Transplantation, Tongji Hospital, Tongji Medical College, Huazhong University of Science and Technology, Wuhan, China

**Keywords:** pegylated granulocyte colony-stimulating factor, stem cell mobilization, myeloid-derived suppressor cells, graft-versus-host disease, allogeneic hematopoietic stem cell transplantation

## Abstract

We compared the effectiveness and safety of pegylated granulocyte colony-stimulating factor (peg-G-CSF) vs. non-peg-G-CSF for hematopoietic stem cell mobilization in allogeneic hematopoietic stem cell transplantation in a real-world setting. We included 136 consecutive healthy donors treated with non–peg-G-CSF (n = 53) or peg-G-CSF (n = 83), and 125 consecutive recipients (n = 42 and 83, respectively) in this study. All harvesting was completed successfully. No significant difference in leukapheresis number and adverse events frequency was observed, nor were there severe adverse events leading to discontinuation of mobilization. The leukapheresis products mobilized by peg-G-CSF had higher total nucleated cells (p < 0.001), monocytic myeloid-derived suppressor cells (p < 0.001), granulocytic myeloid-derived suppressor cells (p = 0.004) and B cells (p = 0.019). CD34+ cells and other lymphocyte subsets (T cells, regulatory T cells, natural killer [NK] cells, etc.) were similar in both apheresis products. Patients who received grafts mobilized by peg-G-CSF exhibited a lower incidence of grade III-IV acute graft-versus-host disease (p = 0.001). The 1-year cumulative incidence of chronic graft-versus-host disease and relapse, 1-year probability of graft-versus-host disease-free relapse-free survival, and overall survival did not differ significantly between subgroups. Our results suggest that collecting allogeneic stem cells after the administration of peg-G-CSF is feasible and safe. Peg-G-CSF mobilized grafts may reduce severe acute graft-versus-host disease compared with non-peg-G-CSF mobilized grafts after allogeneic stem cell transplantation. The beneficial effects of a peg-G-CSF graft might be mediated by increased numbers of monocytic myeloid-derived suppressor cells.

## Introduction

Mobilization with recombinant human granulocyte colony-stimulating factor (G-CSF) followed by leukapheresis has become the standard procedure for obtaining CD34+ peripheral blood stem cells (PBSCs) for hematopoietic stem cell transplantation (HSCT). Some studies optimizing the dosage and schedule of G-CSF have reported an association between G-CSF trough blood levels and mobilization efficacy (1–3). Pegylated recombinant human G-CSF (peg-G-CSF) is a covalently bound conjugate of G-CSF and monomethoxypolyethylene glycol that has a longer elimination half-life than the unconjugated G-CSF because of decreased serum clearance (4). A single injection of peg-G-CSF showed satisfactory efficacy and safety compared with daily G-CSF for reducing neutropenia after chemotherapy (5, 6). Moreover, peg-G-CSF induced the mobilization of CD34 + progenitor cells in animal models (7) and in preliminary human studies involving patients with hematological malignancies as well as healthy donors (8–12). In experimental models, peg-G-CSF showed a modulating impact on both graft-versus-leukemia (GVL) effects (13) and graft-versus-host disease (GVHD) (14) by regulating T cells. Further studies are required to evaluate the mobilization of peg-G-CSF and G-CSF, and to elucidate the mechanisms affecting the recipient’s outcome.

Myeloid-derived suppressor cells (MDSCs) are a heterogeneous cell population that includes immature myeloid cells and the progenitor cells of macrophages, dendritic cells (DCs), monocytes, and neutrophils. MDSCs include two major subpopulations: monocytic MDSCs (M-MDSCs) and granulocytic (polymorphonuclear) MDSCs (G-MDSCs). G-MSDCs share phenotypic and morphologic features with neutrophils, whereas M-MDSCs are similar to monocytes and are characterized by high plasticity. MDSCs are involved in tumor-associated immunosuppression and play immune-regulatory roles in pathologic conditions associated with chronic inflammation or stress (15). Previously, we showed that the accumulation of MDSCs in the graft might contribute to the lower incidence of acute GVHD (aGVHD) in patients and more favorable clinical outcomes after allogeneic HSCT (16). To our knowledge, the relation of MDSC subsets to the mobilization of peg-G-CSF and G-CSF has not been well defined. Therefore, in the present study, we examined the cell type changes, including MDSCs in grafts using different mobilization agents and evaluated the real-world outcomes of the two mobilization agents in patients receiving allogeneic HSCT.

## Methods

### Subjects

The study was approved by the ethics committee of Tongji Hospital, Tongji Medical College, Huazhong University of Science and Technology, in accordance with the Declaration of Helsinki. The medical data of 136 consecutive healthy donors and 125 consecutive patients with hematological diseases who received allogeneic HSCT at our transplantation center from November 2016 to June 2019 were collected. Among the 136 donors, 11 unrelated donors were treated with G-CSF and underwent standard apheresis harvesting at our center. However, the 11 patients received HSCT at other centers, so there were no relevant clinical outcome data. All donors had undergone pre-donation health examinations, including medical history, physical examination, abdominal ultrasound, echocardiography, electrocardiogram, full blood count, blood chemistry analysis, and search for infectious disease markers. Adverse events were classified according to the National Cancer Institute Common Terminology Criteria for Adverse Events, version 5.0 (17).

### Stem Cell Mobilization

G-CSF (Filgrastim, Hangzhou Jiuyuan Gene Engineering Co., Ltd, 2×5 μg/kg per day) or peg-G-CSF (Jinyouli**^®^**, Shijiazhuang Pharmaceutical Group Co., Ltd., single subcutaneous injection: <60 kg, 9 mg; 60–100 kg, 12 mg; >100 kg, 15 mg) were used to mobilize hematopoietic stem cells on day 0. Donor bone marrow and/or peripheral blood cells were collected using standard mobilization protocols. G-CSF mobilized bone marrow (day 3 after G-CSF) and/or peripheral blood progenitor cells (day 4 after G-CSF) were harvested. Peg-G-CSF mobilized bone marrow (day 3 after peg-G-CSF) and/or peripheral blood progenitor cells (day 4 after peg-G-CSF) were harvested. Successful stem cell collection was defined as total nucleated cells > 6 × 10^8^/kg recipient weight and CD34+ cells > 2 × 10^6^/kg recipient weight. If the donor failed to produce a sufficient yield of CD34+ progenitor cells after the first leukapheresis, they were treated with an additional 2×5 μg/kg non-peg-G-CSF.

### Transplant Procedures and GVHD Management

The conditioning regimen was determined according to the patients’ disease type and transplantation type. In short, patients with hematological malignancies were conditioned with cyclophosphamide (Cy)-total body irradiation (TBI)-based or busulfan (Bu)-based myeloablative regimens. Patients with nonmalignant diseases, such as SAA received fludarabine (Flu)/Cy/antithymocyte globulin (ATG) regimens. The day of the stem cell infusions was defined as day 0 when it came to transplantation outcomes. GVHD prophylaxis mainly combined cyclosporine A (CsA), methotrexate (MTX), and mycophenolate mofetil (MMF). Diagnosis and grading of aGVHD were based on the Mount Sinai Acute GvHD International Consortium (MAGIC) criteria (18). Chronic GVHD (cGVHD) was diagnosed and graded using the 2014 National Institutes of Health consensus of cGVHD (19).

### Antibodies and Flow Cytometric Analysis

MDSCs were analyzed using monoclonal antibodies against the following proteins: CD45-PerCP, CD14-APC, CD33-PE, and HLA-DR-FITC (BD Bioscience, San Jose, CA, USA). For each blood sample, background staining and non-leukocytes were corrected by isotype controls and CD45 staining. The percentage of MDSCs in leucocytes was calculated as MDSCs/CD45+ cells. The absolute count of MDSCs in the grafts was calculated as the infused leucocytes per kilogram multiplied by the percentage of MDSCs. The gating strategy for MDSC analysis is shown in **Supplemental Figure 1**. We characterized CD3+CD19− T cells, CD3-CD19+ B cells, CD3+CD4+ T helper cells, CD3+CD8+ cytotoxic T cells, CD4+CD25+CD127dim/− regulatory T cell (Treg), and CD3-CD16+CD56+ natural killer (NK) cells. The cells were stained with the relevant monoclonal antibodies (BD Bioscience), and analyzed on a Becton Dickinson FACS Calibur flow cytometer (BD Biosciences, Franklin Lakes, NJ, USA) and NovoCyte flow cytometer(ACEA Biosciences, San Diego, CA, USA). Data were analyzed with FlowJo 10.0 software (BD Biosciences).

### Outcomes Analysis Standard

The last follow-up for all surviving patients was June 30, 2020. Engraftment was defined as neutrophil counts ≥ 0.5 × 10^9^/L for three consecutive days and platelet counts ≥ 20 × 10^9^/L without transfusion for 7 days. Chimerism analyses were routinely evaluated by PCR of short tandem repeat sequences and/or fluorescence in situ hybridization (FISH) analysis for the X and Y chromosomes (XY-FISH). Donor chimerism status was defined based on previous reports (20) as follows: full donor chimerism, ≥95% donor cells; mixed chimerism, 5–95% donor cells; autologous recovery, ≤5% donor cells. Cytomegalovirus (CMV) viremia was defined as positive results of reverse transcriptase PCR (>1 × 10^3^ copies/mL) in blood. Epstein-Barr virus (EBV) viremia was defined as positive results of reverse transcriptase PCR (>1 × 10^3^ copies/mL or continuous increase) in the blood. CMV disease diagnosis requires clinical symptoms plus CMV documented in tissue by histopathology, virus isolation, rapid culture, immunohistochemistry, or DNA hybridization techniques. Invasive fungal disease (IFD) was defined according to the revised EORTC/MSG criteria (21). Severe bacterial infection was defined as bacteremia or severe tissue infections. The probability of overall survival (OS) was calculated using the Kaplan Meier estimator; death from any cause was considered an event, and surviving patients were censored at last follow-up. GVHD-free relapse-free survival (GRFS) was defined as the absence of grade III to IV aGVHD, cGVHD requiring systemic therapy, relapse, or death (22).

### Statistical Analysis

All data were analyzed using SPSS 22.0 (SPSS Inc., Chicago, IL, USA), GraphPad Prism Version 7 (GraphPad Prism Software Inc., San Diego, CA) and R software 4.0.2. The survival rates were analyzed using the Kaplan-Meier method. Survival differences between groups were estimated by the log-rank test. Cumulative incidences were estimated for GVHD and relapse in which death from any cause was a competing risk for GVHD and relapse. Gray’s test was used in the cumulative incidence analyses. To confirm the outcomes and adjust for potential confounding factors, multivariate Cox models were assessed for the proportional hazards assumption, and interaction terms with covariates were tested. Variables with a p-value that was less than 0.15 in the univariate analyses were included in the Cox model, and the number of variables did not exceed 20% of the size of the valid endpoint. The final multivariate models were constructed using a forward stepwise selection approach. The characteristics among the groups were compared using the Chi-square test for categorical variables and the Mann–Whitney U test for continuous variables. The final model of significance attained ≤ 0.050.

## Results

### Donor Characteristics, Blood Cell Counts, and Safety

Donor characteristics are listed in **Table 1**. The stem cell collection was completed in all donors. About two-thirds of donors yielded adequate numbers of CD34+ cells for transplantation in a single apheresis. The remaining one-third of donors underwent a second apheresis. There was no significant difference in the number of leukapheresis between the G-CSF and peg-G-CSF groups (**Table 1**).

**Table 1:** Donor Characteristics.

Characteristic	G-CSF	PEG-G-CSF	P
Number, n	53	83	
Donor age, median(range) Donor gender, male/female, n WBC,10^9^/L, median(range) Monocyte,10^9^/L, median(range) Peak WBC,10^9^/L, median(range) Peak monocyte,10^9^/L, median(range) Number of leukapheresis, n(%) 1 2	34 (8–59) 40/13 6.8 (3.9–8.5) 0.3 (0.2–0.7) 46.1 (21.5–85.6) 3.5 (1.2–10.3) 33 (62.3) 20 (37.7)	32 (13–56) 61/22 5.6 (3.5–10.3) 0.4 (0.2–0.9) 49.4 (22.3–85.9) 4.3 (1.3–10.6) 56 (67.5) 27 (32.5)	0.458 0.797 0.516 0.610 0.259 0.007 0.534

**Figure 1:**
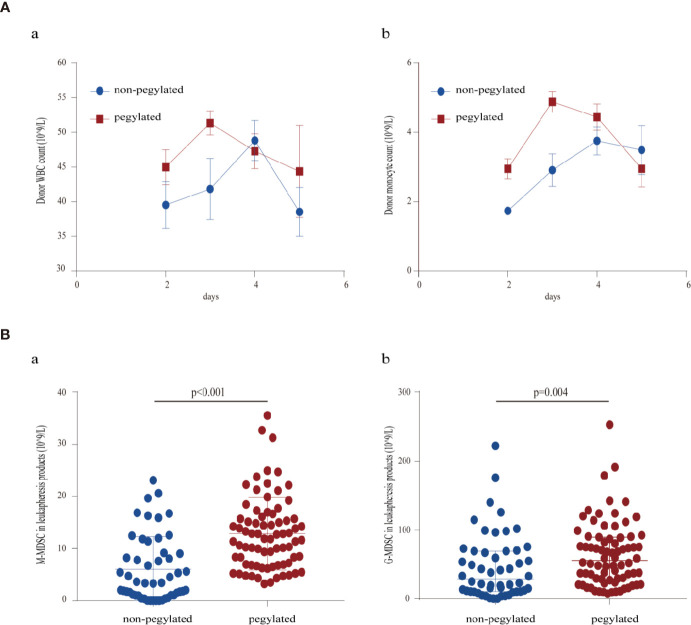
**(A)** Course of donor peripheral blood leukocytes: (a) white blood cell counts (b) monocyte counts; **(B)** MDSC content of first leukapheresis products: (a) M-MDSC in leukapheresis products (b) G-MDSC in leukapheresis products.

Increased white blood cell count and monocyte count in the peripheral blood were observed after mobilization with peg-G-CSF, peaking 3 days after administration, while G-CSF mobilization peaked 4 days after administration (**Figure 1A**).

The adverse events showed no significant difference. Bone pain and headaches were the main adverse effects of cytokine administration as shown in **Table 2**. Flu-like symptoms and changes in serum chemistries (not dynamically assessed) were other common adverse effects. Donors received 1 g loxoprofen orally for pain relief. No donor required narcotic analgesics, or had hyperleukocytosis-related symptoms. No donor discontinued the donation because of adverse events, and all donors underwent successful apheresis as planned. By the end of the follow-up, no donor had obvious abnormalities on medical examination after stem cell collection or hematological malignancies.

**Table 2:** Adverse events.

Subjects with adverse event, n (%)	G-CSF	PEG-G-CSF	p
Any AE during mobilization* AE leading to discontinuation Serious AE after mobilization Hematological malignancies after mobilization Most common AEs during mobilization Bone pain Grade 1–2 Grade 3–4 Headache Grade 1–2 Grade 3–4 Injection site reaction Grade 1–2 Grade 3–4 Flu-like symptoms Grade 1–2 Grade 3–4	49 (92.5) 0 (0.0) 0 (0.0) 0 (0.0) 40 (75.5) 33 (62.3) 7 (13.2) 21 (39.6) 16 (30.2) 5 (9.4) 48 (90.6) 48 (90.6) 0 (0.0) 4 (7.5) 2 (3.8) 2 (3.8)	72 (86.7) 0 (0.0) 0 (0.0) 0 (0.0) 65 (78.3) 50 (60.2) 15 (18.1) 31 (37.3) 21 (25.3) 9 (10.8) 66 (79.5) 66 (79.5) 0 (0.0) 7 (8.4) 5 (6.0) 2 (2.4)	0.300 - - - 0.700 0.814 0.452 0.790 0.532 0.792 0.088 0.088 - 0.853 0.562 0.646

*Adverse events (AEs) that started between the first administration of granulocyte-colony-stimulating factor (G-CSF) and 30 days thereafter were regarded as events that occurred “during” the mobilization period.

### Leukapheresis Products

The characteristics of the leukapheresis products are presented in detail in **Table 3**. The total nucleated cells of the first leukapheresis products from peg-G-CSF-mobilized donors were higher than that of donors mobilized with standard G-CSF. The lymphocytic content of the first leukapheresis products was similar between the two groups. The MDSC content in the peg-G-CSF group was significantly higher than that in the standard G-CSF group as shown in **Figure 1B**. In the second leukapheresis products, however, the cellular content of stem cell products from peg-G-CSF–mobilized donors was similar to that of donors mobilized with standard G-CSF.

**Table 3:** Leukapheresis Products.

Characteristic	G-CSF	PEG-G-CSF	P
1st leukapheresis, median(range)			
Total nucleated cells, 10^9^/L	287.0(130.0,500.6)	343.6(179.9,476.8)	<.001
CD34+ cells, 10^8^/L	8.3(2.1,26.6)	9.6(2.2,31.3)	.166
T+B+NK cells, 10^9^/L	62.7(5.0,129.9)	70.1(3.5,127.0)	.244
CD3+CD19- T cells, 10^9^/L	40.8(3.3,90.0)	46.2(2.4,95.9)	.611
CD3+CD4+ Th cells, 10^9^/L	23.3(1.6,49.3)	24.6 (1.1,53.9)	.627
CD3+CD8+ Ts cells, 10^9^/L	16.4 (1.6,44.0)	16.7(1.2,49.4)	.907
CD3-CD19+ B cells, 10^9^/L	11. 3 (0.4,25.9)	15.1 (0.5,40.9)	.019
CD3-CD16+CD56+ NK cells, 10^9^/L	6. 3(1.3,15.8)	5.8 (1.4,15.7)	.534
CD4+CD25+CD127dim/- Tregs, 10^8^/L	7.1(3.1,22.4)	10.6(4.1,18.7)	.264
Th/Ts	1.35(0.63,3.42)	1.40(0.56,4.05)	.591
M-MDSC, 10^9^/L	3.5 (0.3,23.1)	11.9(3.3,35.6)	<.001
G-MDSC, 10^9^/L	29.0 (0.3,221.7)	55.6(7.4,252.3)	.004
2nd leukapheresis, median(range)			
Total nucleated cells, 10^9^/L	272.2(186.5,480.7)	314.7(182.8,478.1)	.203
CD34+ cells, 10^8^/L	3.7(1.4,13.0)	4.3(1.4,11.6)	.476
T+B+NK cells, 10^9^/L	52.4(30.4,99.9)	51.4(9.1,72.8)	.375
CD3+CD19- T cells, 10^9^/L	37.8(19.9,74.4)	34.8(4.5,46.7)	.339
CD3+CD4+ Th cells, 10^9^/L	19.1(12.4,41.2)	18.4(2.2,34.6)	.708
CD3+CD8+ Ts cells, 10^9^/L	15.4(6.0,26.8)	12.7(2.0,17.5)	.073
CD3-CD19+ B cells, 10^9^/L	8.6(4.2,28.7)	8.7(1.6,19.8)	.586
CD3-CD16+CD56+ NK cells, 10^9^/L	5.1(2.0,12.3)	4.9(1.1,18.4)	.322
Th/Ts	1.46(0.81,3.57)	1.53(0.88,4.39)	.286
M-MDSC, 10^9^/L	7.6(0.3,16.7)	8.9(4.4,35.6)	.743
G-MDSC, 10^9^/L	59.3(0.3,180.9)	126.1(35.9,252.3)	.094

### Recipient Characteristics, Engraftment, and Transplantation Outcome

A total of 125 consecutive patients were enrolled in this cohort. The overall characteristics of the recipient are summarized in **Table 4**.

**Table 4:** Recipient Characteristics.

Characteristic	G-CSF	PEG-G-CSF	P
Number	42	83	
Patient age, median(range)	30 (8, 55)	29 (10, 62)	.782
Patient gender, male/female, n	23/19	51/32	.473
Diagnosis			.445
AML/high-risk MDS, n	21	34	
ALL, n	6	23	
NHL/ANKL/CAEBV, n	7	10	
CML/CMML, n	2	2	
SAA, n	6	14	
Donor age, median(range)	34.5 (8, 59)	32 (13, 56)	.280
Donor gender, male/female, n	31/11	61/22	.970
Donor type, n			<.001
Matched sibling donor	19	21	
Mismatched related donor	14	62	
Matched unrelated donor	9	0	
Donor-recipient sex match, n			.662
Male–male	18	36	
Male–female	13	25	
Female–male	5	15	
Female–female	6	7	
Donor–recipient relation, n			<.001
Sibling–sibling	24	39	
Parent–child	9	27	
Child–parent	0	17	
Others	9	0	
ABO matched, n			.261
Matched	25	44	
Major mismatched	8	19	
Minor mismatched	4	16	
Bidirect mismatched	5	4	
Disease status at HSCT, n*			.910
CR	27	55	
PR	6	13	
Conditioning regimen, n			.759
Bu-Cy-based regimen	31	56	
TBI-Cy-based regimen	5	13	
Flu-Cy-ATG regimen	6	14	
GVHD prophylaxis, n			.008
CsA+MTX	23	22	
CsA+MTX+MMF	14	46	
FK506+MTX+MMF	5	15	

*: n = 101, which did not include SAA and CAEBV.

HSCT, hematopoietic stem cell transplantation; CsA, cyclosporin A; MTX, methotrexate; MMF, mycophenolate mofetil.

The number of total nucleated cells, CD34+ cells, B cells and M-MDSCs transplanted from peg-G-CSF mobilized donors were higher than that from standard G-CSF mobilized donors. These data were shown in detail in **Table 5**. Time to neutrophil and platelet engraftment was documented for 41 patients in the G-CSF cohort (one patient died of Carbapenem-resistant bacteria septicemia 9 days after transplantation) and 83 patients in the peg-G-CSF cohort, respectively, and was not significantly different between the cohorts. Regarding graft failure, platelet engraftment failure occurred in a total of two patients (4.8%) in the G-CSF cohort and two (2.4%) patients in the peg-G-CSF cohort (p = 0.598).

**Table 5:** Engraftment and Transplantation Outcome.

Characteristic	G-CSF	PEG-G-CSF	P
Graft source, n			<0.001
PBSC	27	19	
BM+PBSC	15	64	
Cellular content of infused grafts			
Total nucleated cells, 10^8^/kg	17.5 (5.5–31.8)	23.9(12–0,47.0)	<0.001
CD34+ cells, 10^6^/kg	4.3 (1.2–13.5)	5.2(2.1–13.5)	0.011
CD3+CD19- T cells, 10^8^/kg	2.7 (0.5–7.0)	2.8(1.2–5.3)	0.246
CD3+CD4+ Th cells, 10^8^/kg	1.4 (0.3–3.0)	1.5(0.3–3.7)	0.387
CD3+CD8+ Ts cells, 10^8^/kg	1.0 (0.2–3.2)	1.1(0.4–2.4)	0.509
CD3-CD19+ B cells, 10^8^/kg	0.7 (0.1–1.8)	0.9(0.2–,1.9)	0.004
CD3-CD16+CD56+ NK cells, 10^8^/kg	0.4 (0.2–0.9)	0.4 (0.1–0.9)	0.628
CD4+CD25+CD127dim/− Tregs, 10^6^/kg	3.6 (1.4–10.4)	4.7 (2.1–7.5)	0.309
M-MDSC, 10^6^/kg	32.0 (1.4–126.4)	66.4 (11. 9–298.0)	<0.001
G-MDSC, 10^6^/kg	180.6 (3.1–2073.6)	270.3 (39.9–1812.4)	0.089
Engraftment, days(range)			
Median ANC	11 (8–21)	12 (8–73)	0.563
Median PLT	12 (8–28)	12 (8–46)	0.802
Graft failure, n (%)	2 (4.8)	2 (2.4)	0.598
GVHD, n (%)			
aGVHD	20 (48.8)	38 (45.8)	0.753
III-IV aGVHD	12 (29.3)	6 (7.2)	0.001
cGVHD	15 (36.6)	27 (32.5)	0.654
Moderate-severe cGVHD	7 (17.1)	7 (8.4)	0.153
1-year OS,%	80.5 ± 6.2	83.8 ± 4.1	0.920
1-year GRFS,%	56.1 ± 7.8	70.4 ± 5.2	0.299
1-year relapse rate,%	12.2 ± 0.3	11.1 ± 0.1	0.477
Death reason, n (%)			.656
Relapse	5(11.9)	5(6.0)	
GVHD	2 (4.8)*	6(7.2)^**^	
TA-TMA	2 (4.8)*	1 (1.2)	
IFD	1 (2.4)	1 (1.2)	
Other	1 (2.4)	3 (3.6)	
Infection, n (%)			
Severe bacterial infection	6 (14.6)	13 (15.7)	.881
IFD	6 (14.6)	10 (12.0)	.686
CMV viremia	17 (41.5)	50 (60.2)	.057
CMV Disease	1 (2.4)	6 (7.2)	.424
EBV viremia	35 (85.4)	68 (81.9)	.800

*The two patients underwent TA-TMA before severe GVHD was well controlled.

**Two patients died of GVHD caused by donor lymphocyte infusion after relapse. One patient died of severe chronic liver GVHD.

PBSC, peripheral blood stem cell; M-MDSC, monocytic myeloid-derived suppressor cell; G-MDSC, granulocytic myeloid-derived suppressor cell; ANC, absolute neutrophil count; TA-TMA, transplant-associated thrombotic microangiopathy; IFD, invasive fungal disease.

**Figure 2:**
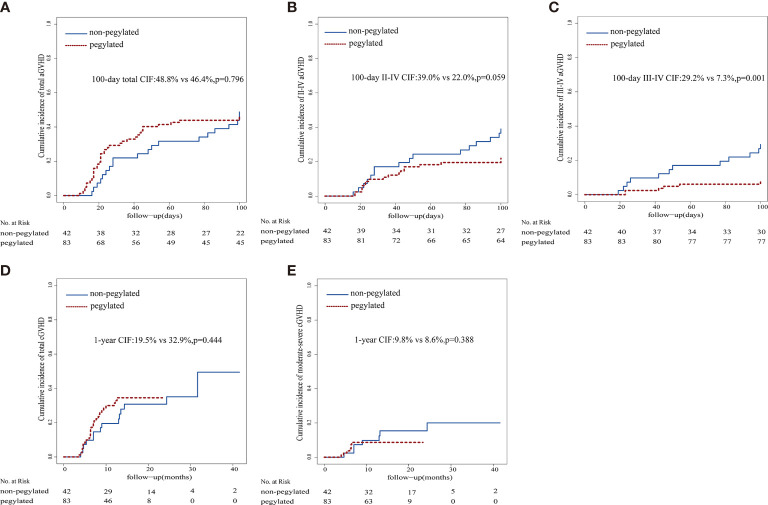
Graft-versus-host disease cumulative incidence. **(A)** aGVHD cumulative incidence, **(B)** II-IV aGVHD cumulative incidence, **(C)** III-IV aGVHD cumulative incidence, **(D)** cGVHD cumulative incidence, **(E)** moderate-severe cGVHD cumulative incidence.

No significant differences in the rates of aGVHD and cGVHD between cohorts were noted, while the proportions of patients with grade III-IV aGVHD in the standard G-CSF cohort were significantly higher than that in the peg-G-CSF cohort. The 100-day cumulative incidence of grade I-IV and III-IV aGVHD and 1-year cumulative incidence of cGVHD were 48.8% vs. 46.4% (p = 0.796), 29.2% vs. 7.3% (p = 0.001), and 19.5% vs. 32.9% (p = 0.444), respectively (**Figure 2**).

**Figure 3:**
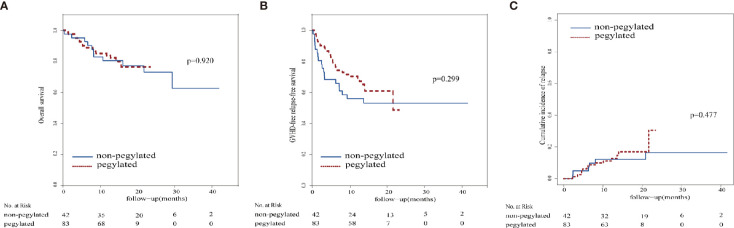
Survival curve and relapse. **(A)** Overall survival, **(B)** Graft-versus-host disease-free relapse-free survival, **(C)** relapse cumulative incidence.

In the standard G-CSF group, 31 patients were alive at the median follow-up of 17.8 months (range, 0.3 - 41.6 months), and the actuarial 1-year OS was 80.5% ± 6.2%. In the peg-G-CSF group, 67 patients were alive at the median follow-up of 13.6 months (range, 1.1 - 23.3 months), and the 1-year OS was 83.8% ± 4.1%. There was no significant difference in the 1-year OS, 1-year GRFS, 1-year probability of relapse, and severe infection between the two groups. The outcomes of the two groups are shown in detail in **Table 5** and **Figure 3**.

### Multivariable Outcome Analysis

**Figure 4:**
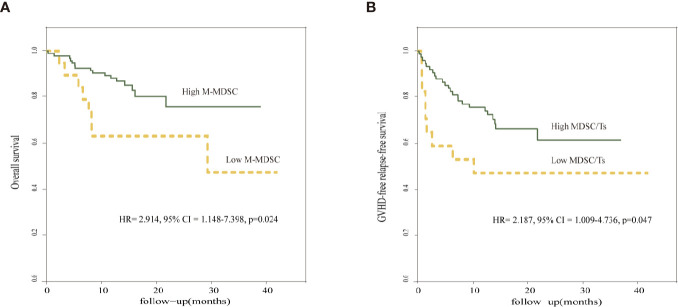
Prognostic factors in multivariate Cox model. **(A)** Comparison of overall survival in relation to M-MDSC. **(B)** Comparison of GRFS in relation to MDSC/Ts.

To confirm the outcomes and adjust for potential confounders, we constructed a multivariate Cox model to test the proportional hazards assumption and interaction terms with covariates. The variables included in the Cox model were selected with univariate analyses. The risk factors calculated for the univariate analysis included donor age, donor type, absolute immune cell counts, and ratio of cell subsets infused in the allograft (**Supplemental Table 1**). Selected numerical variables were categorized according to the respective cutoff points of the receiver operating characteristic (ROC) curve. The respective cutoff points for each variable were calculated to maximize sum of sensitivity and specificity. The total absolute counts of infused M-MDSCs (hazard ratio [HR], 2.914; 95% confidence interval [CI], 1.148–7.398: p = 0.024) in the grafts emerged as an independent factor that influenced OS. The ratio of infused M+G-MDSCs and CD8+ Ts cells in the grafts also affected GRFS (HR, 2.187; 95% CI, 1.009–4.736; *p* = 0.047). There were no significant variables on multivariable analysis for the risk of relapse (**Table 6** and **Figure 4**).

**Table 6:** Multivariate analysis for OS, GRFS and Relapse.

Outcome	Variable	Subtype	Hazard ratio (95%CI)	p value
Overall survival	Patient age	<45-y old ≥45-y old	1.657 (0.520**–**5.280)	0.600
	Donor type	HLA matched HLA mismatched	1.172 (0.448**–**3.069)	0.638
	Th/Ts	<1.16 ≥1.16	1.644 (0.504**–**5.360)	0.296
	M-MDSC,10^6^/kg	>20.82 ≤20.82	2.914 (1.148**–**7.398)	0.024
	MDSC/Th	>0.90 ≤0.90	2.160 (0.788**–**5.918)	0.106
GRFS	Patient age	<45-y old ≥45-y old	1.243 (0.477**–**3.239)	0.674
	Donor type	HLA matched HLA mismatched	1.079 (0.469**–**2.480)	0.897
	Th/Ts	<1.16 ≥1.16	1.648 (0.717**–**3.787)	0.188
	M-MDSC,10^6^/kg	>20.82 ≤20.82	1.146 (0.355**–**3.701)	0.276
	G-MDSC,10^6^/kg	>104.1 ≤104.1	1.230 (0.364**–**4.156)	0.653
	M-MDSC/T	>0.15 ≤0.15	0.889 (0.147**–**5.378)	0.338
	M-MDSC/Ts	>0.39 ≤0.39	1.527 (0.323**–**7.210)	0.382
	MDSC/Ts	>1.38 ≤1.38	2.187 (1.009**–**4.736)	0.047
Relapse	Patient age	<45-y old ≥45-y old	0.717 (0.088**–**5.844)	0.116
	Donor type	HLA matched HLA mismatched	2.318 (0.589**–**9.118)	0.640
	Disease status	CR PR	1.170 (0.316**–**4.335)	0.186
	M-MDSC, 10^6^/kg	>20.82 ≤20.82	1.510 (0.368**–**6.192)	0.209
	G-MDSC,10^6^/kg	>104.1 ≤104.1	1.133 (0.292**–**4.402)	0.198

## Discussion

We designed this study to evaluate the safety and feasibility of stem cell mobilization with peg-G-CSF administered to allogeneic donors. Peg-G-CSF has been shown to be comparable to conventional non-peg-G-CSF in chemotherapy-induced neutropenia and in mobilizing autologous PBSCs (5, 10, 11). Limited preliminary studies (8, 9) have shown that peg-G-CSF is as safe and efficient as standard non-peg-G-CSF in allogeneic donor mobilization. To our knowledge, this is the largest reported series to date providing proof of the comparability of peg-G-CSF and conventional G-CSF in stem cell mobilization. This is also the first study to develop the hypothesis that peg-G-CSF-mobilized grafts might have stronger immunomodulatory properties than G-CSF–mobilized grafts, with a reduced incidence of severe grade III-IV acute GVHD possibly mediated by an increased content of MDSCs.

Peg-G-CSF is the long-acting form of G-CSF. It is a covalent conjugate between a polyethylene glycol molecule and G-CSF, and the cross-linking reaction results in its prolonged duration of action (23, 24). The conventional form of recombinant human G-CSF has a very short half-life and requires multiple daily injections. Typically, it has a half-life of 2–9 h after subcutaneous injection. Conversely, peg-G-CSF, which has a much longer half-life, requires only one injection most of the time, and its half-life ranges from 15–80 h after subcutaneous injection (25). Hence, peg-G-CSF, with its longer bioavailability, might be superior to the conventional form in stem cell mobilization, and donors might highly prefer a single injection of peg-G-CSF compared with multiple injections of non-peg-G-CSF.

In the present study, all donors were harvested successfully. Peg-G-CSF might mobilize hematopoietic cells into the peripheral blood more quickly than non-peg-G-CSF, and this hypothesis was supported by the observation that the maximum leukocyte counts and monocyte counts were found on day 3 in the peg-G-CSF-stimulated donors, which was earlier than that in the non-peg-G-CSF-stimulated donors. The peripheral blood leucocyte and monocyte kinetics observed in our study were similar to that reported previously (9, 26), and the different kinetics we observed could have consequences for leukapheresis scheduling.

Peg-G-CSF might be superior to conventional G-CSF in stem cell mobilization, as peg-G-CSF–stimulated donors had more total nucleated cells of the first leukapheresis products than the G-CSF–stimulated donors (p < 0.001). Due to the superior mobilization with peg-G-CSF compared with standard G-CSF, the predicted rate of failure to mobilize might be similar to or lower than that seen with standard G-CSF. The stem cell products mobilized by peg-G-CSF might have stronger immunomodulatory effects than that mobilized by G-CSF, as the frequency of MDSCs especially M-MDSCs in the pegylated group was much higher than that in the non-pegylated group. Our previous data and many studies have confirmed that MDSCs especially M-MDSCs can prevent GVHD without disabling GVL effect (16, 27–29). G-CSF can induce immune tolerance after HSCT, and G-CSF-induced immune tolerance may be mediated by M/P-MDSCs in allo-HSCT (28, 30). Although the underlying mechanisms of how peg-G-CSF increases MDSC frequency in the graft remain unknown, a previous study has shown that peg-G-CSF mobilized PBSCs showed a gene expression pattern characteristic of immature progenitors and peg-G-CSF mobilized a greater proportion of common myeloid progenitors than unconjugated G-CSF (31). MDSCs are a heterogeneous population of immature myeloid precursors. Dynamic gene expression monitoring that covers the hematopoietic cell subsets mobilized by different G-CSF would be helpful for clarifying this question in further investigations. An interesting finding from our study is that the difference between the two groups disappeared in the second leukapheresis products after conventional G-CSF had been administered in peg-G-CSF mobilized donors. Although this finding might be due to the dynamic changes over time, this finding may also support our hypothesis that the hematopoietic cell kinetics between the groups differ.

Bone pain and headache are the main adverse effects during the administration of mobilization agent. The long half-life of peg-G-CSF could induce the concern of prolonged or excessive leukocytosis, splenic enlargement, and even the potential risk of splenic rupture (9). Although no symptom related to hyperleukocytosis was observed in the present study, periodic abdominal ultrasound should be included in further investigation. Liver function changes, characterized by increases in serum alkaline phosphatase and alanine aminotransferase, have also been reported, and there is no significant difference between peg-G-CSF and G-CSF (9). In the present study, the frequency and intensity of adverse effects in the peg-G-CSF mobilized donors did not appear to be substantially different from that of the donors mobilized with standard G-CSF, which is consistent with available clinical data (8, 25). Further trials should include dynamic monitoring and constant re-assessment of acute and long-term safety.

In our cohort, we observed similar outcomes for OS and GRFS. Furthermore, there were similar relapse rates. Our results for the survival outcomes are, in part, in line with a phase I/II clinical trial, showing satisfactory survival rates in the peg-G-CSF group. Although no comparison for the clinical outcomes between groups was found in that cohort (9), the results of that study corroborate the hypothesis that mobilizing stem cells with peg-G-CSF in normal donors is feasible. The outcomes we report are also consistent with a single center experience by Chanswangphuwana et al., showing similar OS rates (86.7%) (32). GVHD remains one of the major causes of morbidity and mortality in allograft recipients. Recent progress (33–35) in mismatched HSCT has changed the algorithm of donor selection in many transplantation centers. Consequently, haploidentical (haplo)-HSCT has also been increasingly used at our center. Considering the difference in donor type between the G-CSF group and peg-G-CSF group, the latter might have a potentially higher rate of GVHD. However, we observed significantly lower rates of grade III–IV aGVHD, and similar rates of grade I-IV aGVHD and any-grade cGVHD in our cohort. Morris et al. (14) reported that pre-treating donors with a single dose of peg-G-CSF prevented GVHD to a significantly greater extent than standard G-CSF in murines, which was in accordance with our results. In our series, the differences in III–IV aGVHD rates among the two groups may be explained by the M-MDSCs in the infused graft, which has been confirmed to prevent GVHD without disabling the GVL effect. Human MDSCs suppress T cell function through varied mechanisms including L-arginine depletion by arginase1; the generation of inducible nitric oxidase, reactive oxygen species, and indoleamine 2,3-dioxygenase; the release of TGF-β and IL-10; and Treg induction. Otherwise, peg-G-CSF is markedly superior to standard G-CSF for preventing GVHD in animal models following allogeneic stem cell transplantation, due to the generation of IL-10–producing Tregs (14). Mobilization with peg-G-CSF results in enhanced expansion of tolerogenic antigen-presenting cells and the augmentation of Treg activity that in turn can reduce GVHD (36). Modification of G-CSF by pegylation of the native cytokine results in the expansion and activation of donor invariant NK T cells, which significantly augment CD8+ T-cell-mediated cytotoxicity and GVL effects after transplantation (13, 37). Consequently, peg-G-CSF further separates GVHD and the GVL effect. High levels of M-MDSCs among the grafts can also reduce the incidence of cGVHD (28), which could partly explain our finding that although B cells in the infused grafts were significantly different, the incidence of cGVHD between groups was similar. Considering that both donor T and B cells play an essential role in the development of cGVHD and that the follow-up was short, further monitoring is needed to draw a more accurate conclusion.

We acknowledge important limitations of our study, which are mainly related to the relatively small sample size and single-center, retrospective nature, including the short median follow-up of 13.6 months for the peg-G-CSF group and the lack of the aforementioned data (donor blood biochemistry test etc.). The function test of MSDC in different harvests was not performed. In our future research, we will supplement relevant experiments to further elucidate the underlying mechanism. Experiences with peg-G-CSF in healthy donors are still very limited. A prospective randomized clinical trial (ChiCTR2000032370) is ongoing at our center to yield better insight into peg-G-CSF mobilization and to provide more valid data. Although our data support the premise that the higher MDSCs in grafts mobilized by peg-G-CSF are associated with lower risk of severe aGVHD, the complete mechanism remains to be explored by more functional assays in the future.

In conclusion, although the mobilization effectiveness of peg-G-CSF appears comparable to that of G-CSF, less discomfort after administration and the lower incidence of severe aGVHD represent arguments for the use of peg-G-CSF. Peg-G-CSF mobilized grafts might ameliorate severe aGVHD by enriching M-MDSCs in the graft. These findings require validation in large prospective randomized trials and real world data.

## Data Availability Statement

The original contributions presented in the study are included in the article/**Supplementary Material**. Further inquiries can be directed to the corresponding author.

## Ethics Statement

The studies involving human participants were reviewed and approved by the ethics committee of Tongji Hospital, Tongji Medical College, Huazhong University of Science and Technology. Written informed consent to participate in this study was provided by the participants’ legal guardian/next of kin.

## Author Contributions

All authors contributed to the study conception and design. Material preparation, data collection and analysis were performed by LLi, JY, and YL. The first draft of the manuscript was written by LLi and all authors commented on previous versions of the manuscript. All authors contributed to the article and approved the submitted version.

## Funding

This research was supported by grants from the National Natural Science Foundation of China (grants 81570163, 81873446, 81873427, 81700174, 81400147).

## Conflict of Interest

The authors declare that the research was conducted in the absence of any commercial or financial relationships that could be construed as a potential conflict of interest.

## Supplementary Material

Supplementary Figure 1 Gate strategy of MDSCs subsets **(A)** General gate on FSC and SSC **(B)** CD45+ cells **(C)** HLA-DR^-/low^ cells **(D)** CD33+CD14+, M-MDSC; CD33^dim^, G-MDSC.
