# Supplementary figures and images for: Allogeneic Hematopoietic Stem Cell Transplantation Mobilized With Pegylated Granulocyte Colony-Stimulating Factor Ameliorates Severe Acute Graft-Versus-Host Disease Through Enrichment of Monocytic Myeloid-Derived Suppressor Cells in the Graft: A Real World Experience
Source: Front Immunol. 2021 Apr 12;12:621935. doi: 10.3389/fimmu.2021.621935 (PMC8072473; doi:10.3389/fimmu.2021.621935)

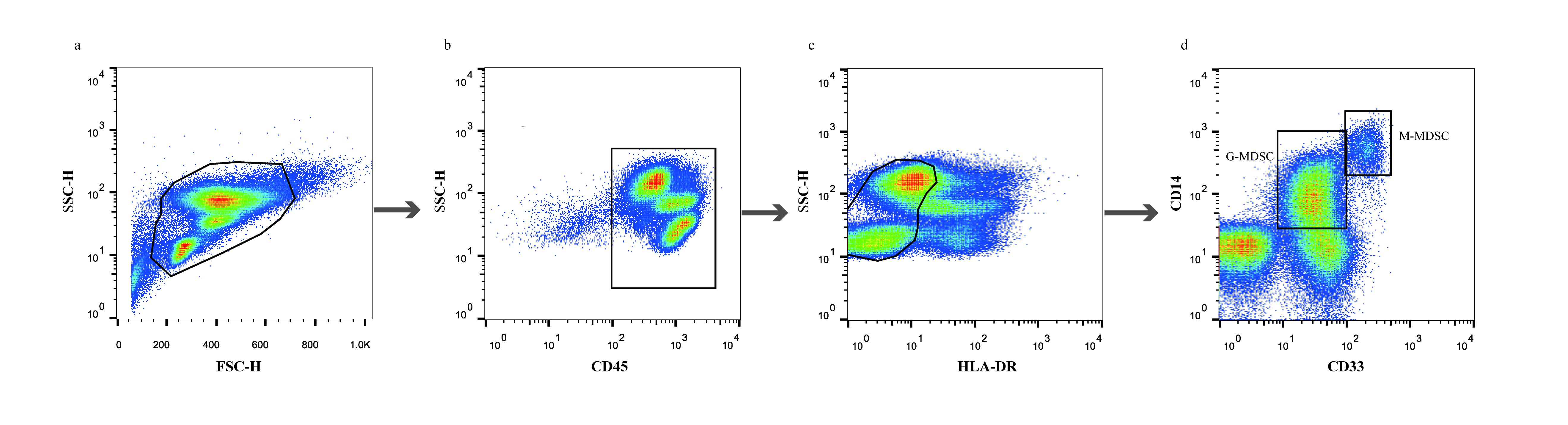

Supplement: Supplementary Figure 1 — Gate strategy of MDSCs subsets (A) General gate on FSC and SSC (B) CD45+ cells (C) HLA-DR-/low cells (D) CD33+CD14+, M-MDSC; CD33dim, G-MDSC. [file Image_1.tif]
