# Supplementary material for: Allogeneic Hematopoietic Stem Cell Transplantation Mobilized With Pegylated Granulocyte Colony-Stimulating Factor Ameliorates Severe Acute Graft-Versus-Host Disease Through Enrichment of Monocytic Myeloid-Derived Suppressor Cells in the Graft: A Real World Experience
Source: Front Immunol. 2021 Apr 12;12:621935. doi: 10.3389/fimmu.2021.621935 (PMC8072473; doi:10.3389/fimmu.2021.621935)
Supplement: Supplementary file 2 [file Table_1.docx]

**Supplemental Table 1. Univariate analysis for OS，GRFS and relapse.**

| Variable | Subtype | OS(p value) | GRFS(p value) | Relapse(p value) |
| --- | --- | --- | --- | --- |
| ABO incompatibility  Mobilization agent  Patient age  Donor type  Donor Age  Donor gender  M-MDSC in graft  G-MDSC in graft  MDSC in graft  Th/Ts  M-MDSC/T  M-MDSC/Th  M-MDSC/Ts  G-MDSC/T  G-MDSC/Th  G-MDSC/Ts  MDSC/T  MDSC/Th  MDSC/Ts | Match vs. Mismatch  non-pegylated G-CSF vs. pegylated G-CSF  <45-y old vs. ≥45-y old  HLA matched vs. HLA mismatched  <45-y old vs. ≥45-y old  Male vs. Female  >20.82*10^6^/kg vs. ≤20.82*10^6^/kg  >104.1*10^6^/kg vs. ≤104.1*10^6^/kg  >152.5*10^6^/kg vs. ≤152.5*10^6^/kg  <1.16 vs. ≥1.16  >0.15 vs. ≤0.15  >0.25 vs. ≤0.25  >0.39 vs. ≤0.39  >0.46 vs. ≤0.46  >0.95 vs. ≤0.95  >0.95 vs. ≤0.95  >0.54 vs. ≤0.54  >0.90 vs. ≤0.90  >1.38 vs. ≤1.38 | 0.466  0.920  0.920  0.732  0.461  0.519  0.035  0.218  0.290  0.084  0.179  0.242  0.134  0.147  0.205  0.357  0.130  0.029  0.126 | 0.350  0.299  0.895  0.665  0.274  0.959  0.061  0.132  0.237  0.136  0.109  0.137  0.131  0.180  0.492  0.204  0.178  0.138  0.042 | 0.462  0.477  0.211  0.059  0.852  0.284  0.977  0.125  0.877  0.309  0.405  0.270  0.880  0.994  0.457  0.654  0.846  0.722  0.927 |
