# Supplementary material for: Allogeneic Hematopoietic Stem Cell Transplantation Mobilized With Pegylated Granulocyte Colony-Stimulating Factor Ameliorates Severe Acute Graft-Versus-Host Disease Through Enrichment of Monocytic Myeloid-Derived Suppressor Cells in the Graft: A Real World Experience
Source: Front Immunol. 2021 Apr 12;12:621935. doi: 10.3389/fimmu.2021.621935 (PMC8072473; doi:10.3389/fimmu.2021.621935)
Supplement: Supplementary file 3 [file Table_2.docx]

**Supplemental Table 1. Univariate analysis for OS，GRFS and relapse.**

| Variable | Subtype | OS(p value) | GRFS(p value) | Relapse(p value) |
| --- | --- | --- | --- | --- |
| ABO incompatibility  Mobilization agent  Patient age  Disease status  Donor type  Donor Age  Donor gender  M-MDSC in graft  G-MDSC in graft  MDSC in graft  Th/Ts  M-MDSC/T  M-MDSC/Th  M-MDSC/Ts  G-MDSC/T  G-MDSC/Th  G-MDSC/Ts  MDSC/T  MDSC/Th  MDSC/Ts | Match vs. Mismatch  non-pegylated G-CSF vs. pegylated G-CSF  <45-y old vs. ≥45-y old  CR vs. PR  HLA matched vs. HLA mismatched  <45-y old vs. ≥45-y old  Male vs. Female  >20.82*10^6^/kg vs. ≤20.82*10^6^/kg  >104.1*10^6^/kg vs. ≤104.1*10^6^/kg  >152.5*10^6^/kg vs. ≤152.5*10^6^/kg  <1.16 vs. ≥1.16  >0.15 vs. ≤0.15  >0.25 vs. ≤0.25  >0.39 vs. ≤0.39  >0.46 vs. ≤0.46  >0.95 vs. ≤0.95  >0.95 vs. ≤0.95  >0.54 vs. ≤0.54  >0.90 vs. ≤0.90  >1.38 vs. ≤1.38 | .466  .920  .920  .105  .732  .461  .519  .035  .218  .290  .084  .179  .242  .134  .147  .205  .357  .130  .029  .126 | .350  .299  .895  .278  .665  .274  .959  .061  .132  .237  .136  .109  .137  .131  .180  .492  .204  .178  .138  .042 | .462  .477  .211  < .001  .059  .852  .284  .977  .125  .877  .309  .405  .270  .880  .994  .457  .654  .846  .722  .927 |
